# Genetic Control of Immune Response and Susceptibility to Infectious Diseases

**DOI:** 10.1155/2014/796073

**Published:** 2014-02-13

**Authors:** Enrique Medina-Acosta, Helder Takashi Imoto Nakaya, Alessandra Pontillo, Regina Célia de Souza Campos Fernandes

**Affiliations:** ^1^Department of Biotechnology, Center for Biosciences and Biotechnology, State University of Norte Fluminense Darcy Ribeiro, 28013-602 Campos dos Goytacazes, RJ, Brazil; ^2^Emory Vaccine Center, Yerkes National, Primate Research Center, Emory University, Atlanta, GA 30329, USA; ^3^Laboratory of Immunogenetics, Department of Immunology, Institute of Biomedical Sciences, University of São Paulo, 05508-900 São Paulo, SP, Brazil; ^4^Department of Pediatrics, School of Medicine of Campos, Álvaro Alvim School Hospital, 28035-211 Campos dos Goytacazes, RJ, Brazil

Both genetic and nongenetic variables are known to impact, in magnitude and breadth, immune responses to infectious disease agents. Discovery and validation of genetic determinants in hosts and pathogens are crucial to better understand the basis of susceptibility to and control of infectious diseases. The interplay of these conjoined yet opposed multiple, varying factors results in an impressive dynamic phenotypically diversity in hosts. The precise mechanisms that underlie the observed interindividual variation in control of, resistance, and/or susceptibility to infectious diseases are not completely understood. 

This special issue gathers three original research papers and five reviews that aimed at stimulating future research efforts towards medical and veterinary applications of genetic variation with well-defined genetic roles in host immune response and susceptibility to infectious diseases. Overall, this area of research has been dominated by population genetics on candidate genes through small scale interindividual susceptibility association studies. The observed interindividual variation is due, in part, to risk-modifying polymorphisms (rare and common) in agonist and antagonist genes of the innate and adaptive immune responses. There is robust evidence for association with control to or decrease susceptibility to infectious agents or disease progression for some host gene variants. The perspective is that the field will benefit from both small-scale replica and genomewide association studies (GWAS), firstly to validate the statistical strength of observed associations in different population backgrounds and secondly to discover significant associations with newly investigated variants with translational potential into practical tests.

A.  V. Marangon et al. looked into the association of HLA class I, HLA class II, and *KIR* genes as well as single nucleotide polymorphisms (SNP) in cytokine genes (*IFNG*,* IL6*, *IL10*, *TGFB1*,* and TNF*) with risk for human-papillomavirus- (HPV-) related cervical disease in two groups of subjects: 79 unrelated admixed Brazilian women who tested positive for carcinogenic high risk HPV and presented with cervical intraepithelial neoplasia grade 3 (CIN3), and 150 HPV-negative women from the same ethnicity. The most significant findings of this study are (i) none of the fourteen *KIR* genes, the *KIR* pseudogene, HLA class I/II alleles (or haplotype combination thereof) was significantly associated with HPV-related cervical disease; (ii) the HLA-A*02-B*51 haplotype was associated with resistance to HPV-related cervical disease; (iii) the *IFNG* +874A/A genotype was associated with susceptibility to HPV infection.

Z.-Q. Feng et al. tested whether the retinoic acid-induced gene I (RIGI)-like receptors (RLRs)-viral detection and antiviral response pathways against RNA viruses are involved in resistance to Marek's disease virus (MDV), a DNA virus, in chickens, and whether resistance to infection is affected by the genetic background of chickens. The authors experimentally infected two-chicken breeds (economic line-AA broilers and native Erlang mountainous chickens) to comparatively monitor patterns of RNA expression of melanoma differentiation associated gene 5 (*MDA-5*), interferon regulatory transcription factor 3 (*IRF-3*), *IFN-*α*,* and *IFN-*β** by real-time PCR on total cDNAs from various tissues. The most significant findings of this study are (i) the Erlang mountainous chicken breed, but not the economic line-AA breed, survived MDV infection with slight increased control of viral loads; (ii) RNA expression for *MDA-5*, *IRF-3*, *IFN-*α**, and *IFN-*β** increased to different magnitudes following infection in either breed, but opposing magnitudes were observed in different tissues. The authors' perspective is that RLR-mediated antiviral pathway is involved in detection and response against MDV.

A.  C. Leandro et al. interrogated *IFNG*+874*T/A and *NOS2A*-954*G/C SNPs in a case-control (*n* = 172/*n* = 179) study to estimate their role on susceptibility to tuberculosis and to determine whether those variants impact the levels of nitrite and nitric oxide radical in serum. The most significant finding of this study is that neither allele variant nor a combination thereof was significantly associated with either risk of developing tuberculosis or differences in secretion levels of nitric oxide radicals in the admixed Brazilian population subset studied.

C.  M. Ayo et al. lent a comprehensive overview about the associations between polymorphisms in HLA class I/II,* KIR*, cytokine (*CCL2*,* CXCL9*,* IFNG*,* IL1B*,* IL12B*,* IL4*,* IL6*,* IL10*,* LTA*, *TGFB1, and TNF*), cytokine receptor (*CCR5*,* IL4R*) genes and the splicing factor *BAT1 *(DDX39B) gene and the occurrence, severity, and clinical forms of Chagas disease (American trypanosomiasis). A commendable contribution of this review is the reporting of statistical significance (*P* values) of the associations from the various studies observed between HLA alleles and haplotypes and the different clinical forms of the disease (chronic Chagas cardiomyopathy, digestive form, and mixed and combinations thereof). Despite the many linkage and association studies carried out to discover host genetic variants involved in immunopathogenesis of Chagas disease, causality of susceptibility remains a challenge.

L.  R. Jarduli et al. contributed with a resourceful review about the roles of HLA, *KIR,* and *MICA* genes as well as common polymorphisms in pro- and anti-inflammatory cytokine (*IFNG*,* IL1B*,* IL12B*,* IL4*,* IL6*,* IL10*,* TNF*,* and LTA*) and cytokine receptor (*IFNGR1*,* IL12RB1, and IL12RB2*), *BAT1 *and the* BTNL2 *(butyrophilin-like 2 HLA class II associated) gene in resistance or susceptibility to infection by *Mycobacterium leprae* and the clinical course and varying manifestations of Leprosy. A praiseworthy aspect of this review is the scope by clinical forms of the disease (multibacillary, lepromatous, borderline, tuberculoid, and erythema nodosum leprosum), with the inclusion of statistical significance (*P* values) for the associations observed in twin studies, segregation analyses, family-based linkage and association studies, candidate gene association studies, and, most recently, GWAS. Interestingly, some HLA and *KIR* gene combinations rendered favorable or unfavorable interactions, with opposing (activation or inhibition) effects on NK cells and infected host cells, which underscores the interplay of host genetic factors in pathogenesis.

F. Celsi et al. bestowed an enlightened account of the gene expression regulatory plans devised by human immunodeficiency virus type 1 (HIV-1) to escape the immune response against infected cells, given emphasis on the interplay of classical class I HLA-C and nonclassical class I HLA-G alleles and genotypes associated with effective control of viral replication and with slow progression to AIDS. The realization that genetic background differences (i.e., ethnicity) in HLA-G (i.e., HLA-G*0105N) allele distribution may influence the outcome(s) (protection/susceptibility) of association studies urgently calls for replica studies. The authors further assessed the newly discovered small noncoding RNAs (miRNAs) encrypted in the host genome for downregulating HLA-C and HLA-G gene expression through RNA interference at the 3′ untranslated regions (UTR). The possible effects of SNPs at HLA-C and HLA-G 3′ UTRs on miRNA binding were discussed as well as the prospects of using miRNA expression signatures as a biomarker of disease progression. The authors' perspective is that estimating the impact of such regulatory mechanisms on HIV-1 infection, replication, and progression to AIDS will uncover novel therapeutic targets. The prospects of existence of counteracting miRNA encrypted (putatively) in the HIV-1 genome pave the way for unprecedented approaches.

V. C. Vieira and M. A. Soares imparted a fresh appraisal of the DNA and/or RNA sequence editing dependent or independent roles of the human APOBEC family of cytidine deaminases in innate immune inhibition of viral infections (including EBV, HBV, HCV, HIV-1, HPV, HSV-1, and HTLV) as well as of the counteracting viral strategies ensued. A praiseworthy aspect of this review is the comprehensive and insightful comparison of studies on polymorphisms in *APOBEC3* genes and their associations with susceptibility to viral infections. The authors' perspective is that enhancing APOBEC3 activity constitutes a novel approach for antiviral drug development, notwithstanding the realization that APOBEC3-mediated hypermutating viral DNA/RNA may increase viral genetic diversification and, thus, emergence of viral escape and/or drug-resistant variants.

J. Malogajski et al. summated the evidence for high translational potential of basic host genomic and genetic studies for three sexually transmitted agents (HIV-1, *Chlamydia trachomatis*, and HPV) into applications of public health and care including diagnosis, treatment, and prevention of diseases. The authors asserted that there is considerable lagging between discovery of functional genetic polymorphisms and effective introduction of practical applications, which points to fertile research grounds in the areas of pharmacogenomics and personalized medicine. In fact, no market-ready application involving genetic/genomic tests for the diseases caused by these infectious agents is, at present, available. The prospects of introducing routine screening tests for epigenetic (DNA methylation) markers, known to be associated with high grade cervical intraepithelial neoplasia and cervical cancer, meet the expectation of significantly reducing the number of unnecessary referrals to gynecologists. Similarly, bringing in *TLR4* SNP typing and *TLR2* haplotyping for identification of variants or haplotypes linked to either increased risk of tubal pathology or protection from tubal pathology, respectively, is expected to provide for a more accurate diagnosis of *Chlamydia trachomatis* causing subfertility.

We expect that the papers in this special issue will hasten perceptiveness towards discovery and application of genetic variants in biotechnology, diagnostics, and treatment of infectious diseases of both man and animals.

*Enrique Medina-Acosta* *Helder Takashi Imoto Nakaya* *Alessandra Pontillo* *Regina Célia de Souza Campos Fernandes*

